# Combined Clinical Audits and Low-Dose, High-frequency, In-service Training of Health Care Providers and Community Health Workers to Improve Maternal and Newborn Health in Mali: Protocol for a Pragmatic Cluster Randomized Trial

**DOI:** 10.2196/28644

**Published:** 2021-12-10

**Authors:** David Zombre, Jean-Luc Kortenaar, Farhana Zareef, Moussa Doumbia, Sekou Doumbia, Fadima Haidara, Katie McLaughlin, Samba Sow, Zulfiqar A Bhutta, Diego G Bassani

**Affiliations:** 1 Centre for Global Child Health The Hospital for Sick Children Toronto, ON Canada; 2 Dalla Lana School of Public Health University of Toronto Toronto, ON Canada; 3 Centre for Vaccine Development Bamako Mali; 4 Centre for Excellence in Women and Child Health and Institute for Global Health and Development The Aga Khan University Karachi Pakistan; 5 Department of Paediatrics University of Toronto Toronto, ON Canada

**Keywords:** perinatal mortality, low dose high frequency training, maternal and newborn health outcomes, Mali

## Abstract

**Background:**

Although most births in Mali occur in health facilities, a substantial number of newborns still die during delivery and within the first 7 days of life, mainly because of existing training deficiencies and the challenges of maintaining intrapartum and postpartum care skills.

**Objective:**

This trial aims to assess the effectiveness and cost-effectiveness of an intervention combining clinical audits and low-dose, high-frequency (LDHF) in-service training of health care providers and community health workers to reduce perinatal mortality.

**Methods:**

The study is a three-arm cluster randomized controlled trial in the Koulikoro region in Mali. The units of randomization are each of 84 primary care facilities. Each trial arm will include 28 facilities. The facilities in the first intervention arm will receive support in implementing mortality and morbidity audits, followed by one-day LDHF training biweekly, for 6 months. The health workers in the second intervention arm (28 facilities) will receive a refresher course in maternal neonatal and child health (MNCH) for 10 days in a classroom setting, in addition to mortality and morbidity audits and LDHF hands-on training for 6 months. The control arm, also with 28 facilities, will consist solely of the standard MNCH refresher training delivered in a classroom setting. The main outcomes are perinatal deaths in the intervention arms compared with those in the control arm. A final sample of approximately 600 deliveries per cluster was expected for a total of 30,000 newborns over 14 months. Data sources included both routine health records and follow-up household surveys of all women who recently gave birth in the study facility 7 days postdelivery. Data collection tools will capture perinatal deaths, complications, and adverse events, as well as the status of the newborn during the perinatal period. A full economic evaluation will be conducted to determine the incremental cost-effectiveness of each of the case-based focused LDHF hands-on training strategies in comparison to MNCH refresher training in a classroom setting.

**Results:**

The trial is complete. The recruitment began on July 15, 2019, and data collection began on July 23, 2019, and was completed in November 2020. Data cleaning or analyses began at the time of submission of the protocol.

**Conclusions:**

The results will provide policy makers and practitioners with crucial information on the impact of different health care provider training modalities on maternal and newborn health outcomes and how to successfully implement these strategies in resource-limited settings.

**Trial Registration:**

ClinicalTrials.gov NCT03656237; https://clinicaltrials.gov/ct2/show/NCT03656237

**International Registered Report Identifier (IRRID):**

DERR1-10.2196/28644

## Introduction

### Background

Mothers and their babies are at the highest risk of death during delivery and during the first week postpartum [1]. Even though 59% of births in sub-Saharan African countries now occur in health facilities [2], in 2018, approximately 2.476 million babies died during the first 28 days of life, with approximately 1 million dying during the first day of life and almost another 1 million within the first week [3,4]. In Mali, an estimated 67 stillbirths [5] and an additional 75 newborn deaths [6] occur on average every day; the predominant causes are birth asphyxia, prematurity, and sepsis [7]. The maternal mortality ratio in Mali is 562 per 100,000 live births [3], almost 2.7-fold the global rate [3]. Most of these deaths are because of factors related to the place of delivery and quality of care [4,8]. In particular, the lack of appropriately trained staff in facilities, delays in referrals, and inadequate supplies and equipment are evident in many resource-poor settings [9]. This suggests that high-quality care, especially during late pregnancy, childbirth, and the early newborn period, are essential [10], and simple evidence-based interventions are likely to meaningfully reduce the number of maternal deaths, stillbirths, and early neonatal deaths [11,12].

Traditional capacity-building models for training health care providers, delivered through off-site classroom-style lectures or directed reading, are still the norm in low- and middle-income countries [8,13,14]. However, to disrupt the delivery of services, these approaches have been found to have limited (if any) impact on learning outcomes, and any knowledge and skills gained are neither transferred to other coworkers at the facility nor translated into improved provider practice and performance [13,15]. Moreover, there is no evidence that these methods contribute to knowledge retention after training, and limited conclusions can be drawn about their long-term impact on measures of performance in terms of service quality and maternal and newborn outcomes. A review of intervention studies aimed at improving the performance of health workers in low- and middle-income countries has suggested that problem-based learning and competence-based learning [15], including supervision and audits with feedback [13,14], along with brief team learning sessions within the workplace (health facility), using simulation, followed by simulation-based practice and feedback, can improve learning outcomes and provider performance compared with training approaches that do not include subsequent practice [13,16].

Over the last few years, an increasing number of African countries have implemented competency-based training of health care providers with the goal of improving maternal and child health outcomes and reducing perinatal mortality [17-19]. Low-dose, high-frequency (LDHF) training is a type of competency-based training that promotes maximal retention of clinical knowledge, skills, and attitudes through short, targeted, simulation-based learning activities, delivered during several in-service sessions and reinforced with structured practice sessions [20].

In that vein, in response to a World Health Organization report on health systems and policy indicators documenting that Mali did not have a policy on maternal, perinatal, and neonatal death audit (World Health Organization 2014), Mali’s Ministry of Public Health and Hygiene made it mandatory to report maternal, perinatal, and neonatal deaths and adverse events within 48 hours of their occurrence, through the Integrated Disease Surveillance and Response Network, and to convene an audit of the events within 15 days of the notification.

Mali’s national directive to implement facility-based maternal and perinatal morbidity and mortality audits offers an opportunity to leverage it and target training on skills and competency gaps identified during audits. However, to the best of our knowledge, the potential of using facility-based maternal and perinatal morbidity and mortality audits to identify areas that require attention for subsequent LDHF hands-on training has not yet been exploited. Moreover, even though LDHF hands-on training has demonstrated a significant positive impact on maternal and early neonatal outcomes, including reduced rates of fresh stillbirth and first-day mortality [17,21], retained placenta, and postpartum hemorrhage [19]. Limited conclusions can be drawn about the impact of LDHF hands-on training on maternal and neonatal care practices during intrapartum and postpartum periods and other perinatal outcomes, especially 7-day mortality and morbidity. There is very little known about the cost-effectiveness and the processes of implementation of LDHF training [22,23].

### Objective

This study proposes a strategy to leverage maternal and perinatal morbidity and mortality audits being implemented in Mali, as a basis for identifying gap areas that can be addressed by a case-based focused LDHF on-site training strategy within primary health care–level facilities (Centre de Santé Communautaire [CSCOM]). These trainings will also target community-based causes of mortality and morbidity in health facilities. The trial will assess the effectiveness of LDHF hands-on training informed by maternal and neonatal mortality audits in reducing perinatal mortality and in increasing adoption of key maternal and newborn care practices during the delivery and the postpartum period. Secondary objectives include assessment of the implementation process and the cost-effectiveness of the intervention.

## Methods

### Study Setting

The study will be conducted in 4 districts in the Koulikoro region, Mali. The study area was selected as it is part of a larger project implemented by the Canadian Red Cross in collaboration with the Malian Red Cross (Croix-Rouge Malienne). This project is an integrated 4-year (2016-2020) maternal neonatal and child health (MNCH) intervention that targets 6 districts across the Koulikoro and Sikasso regions and is funded by the Government of Canada. The project supports the primary and secondary health facilities (CSCOMs and Centre de Santé de Référence [CSREF]—referral health centers) to improve the quality of their MNCH services and strengthen the health management information system. At the community level, the Malian Red Cross has a strong presence because of the training and support of local community health workers and volunteers for MNCH-related activities at remote integrated management of childhood illness sites.

The Koulikoro region is the second-most-western region of Mali. It covers an area of 90,120 square kilometers. With an estimated population in 2017 of 3.1 million and a density of 26.8 people per square kilometer. The Koulikoro region is separated into 7 districts: Kati, Kangaba, Koulikoro, Kolokani, Nara, Banamba, and Dioïla. The Canadian Red Cross and Malian Red Cross program encompasses the latter five. Of these, 4 districts (Koulikoro, Kolokani, Banamba, and Dioïla) will be included in this study.

### Trial Design

This pragmatic (effectiveness) cluster randomized controlled trial will be implemented in 84 community health facilities (CSCOM) in the Koulikoro region, Mali. Facilities will be randomly allocated into either of two intervention arms or the control arm.

- Intervention arm 1: audit and feedback with LDHF hands-on training and 6 months follow-up.
- Intervention arm 2: MNCH refresher training and audit, and feedback with LDHF hands-on training and 6 months follow-up.
- Control arm: allocated to receive MNCH refresher training and follow-up.

### Cluster Size and Selection

Community health facilities, including community health workers serving the catchment area, are the units of randomization (clusters). Although there are some differences in the services provided at these facilities according to the baseline facility assessment conducted in December 2016, only those health facilities providing basic obstetric care with at least one health care provider trained in the active management of the third stage of labor (AMTSL), essential newborn care (ENC), perinatal care (PNC), and those who had not yet started implementing the audits are considered for inclusion in the study. All facility deliveries (both mother and newborn) within study clusters and live home births referred to a facility within the perinatal period are included in the trial, subject to informed consent. In that respect, a master list of 84 facilities meeting the inclusion criteria within the 4 districts will be generated.

### Randomization

**Figure 1:**
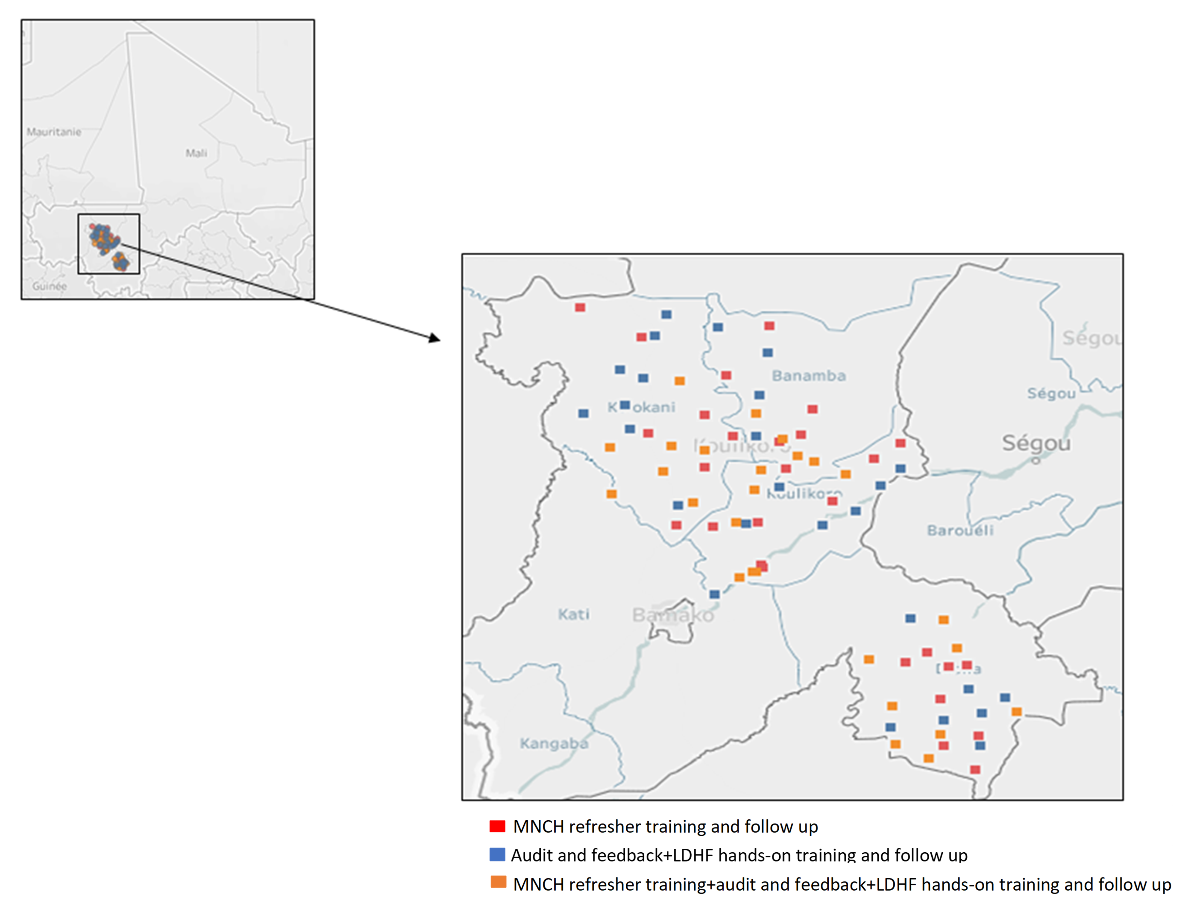
Geographical location of health districts and health facilities under study. LDHF: low-dose, high-frequency; MNCH: maternal neonatal and child health.

**Figure 2:**
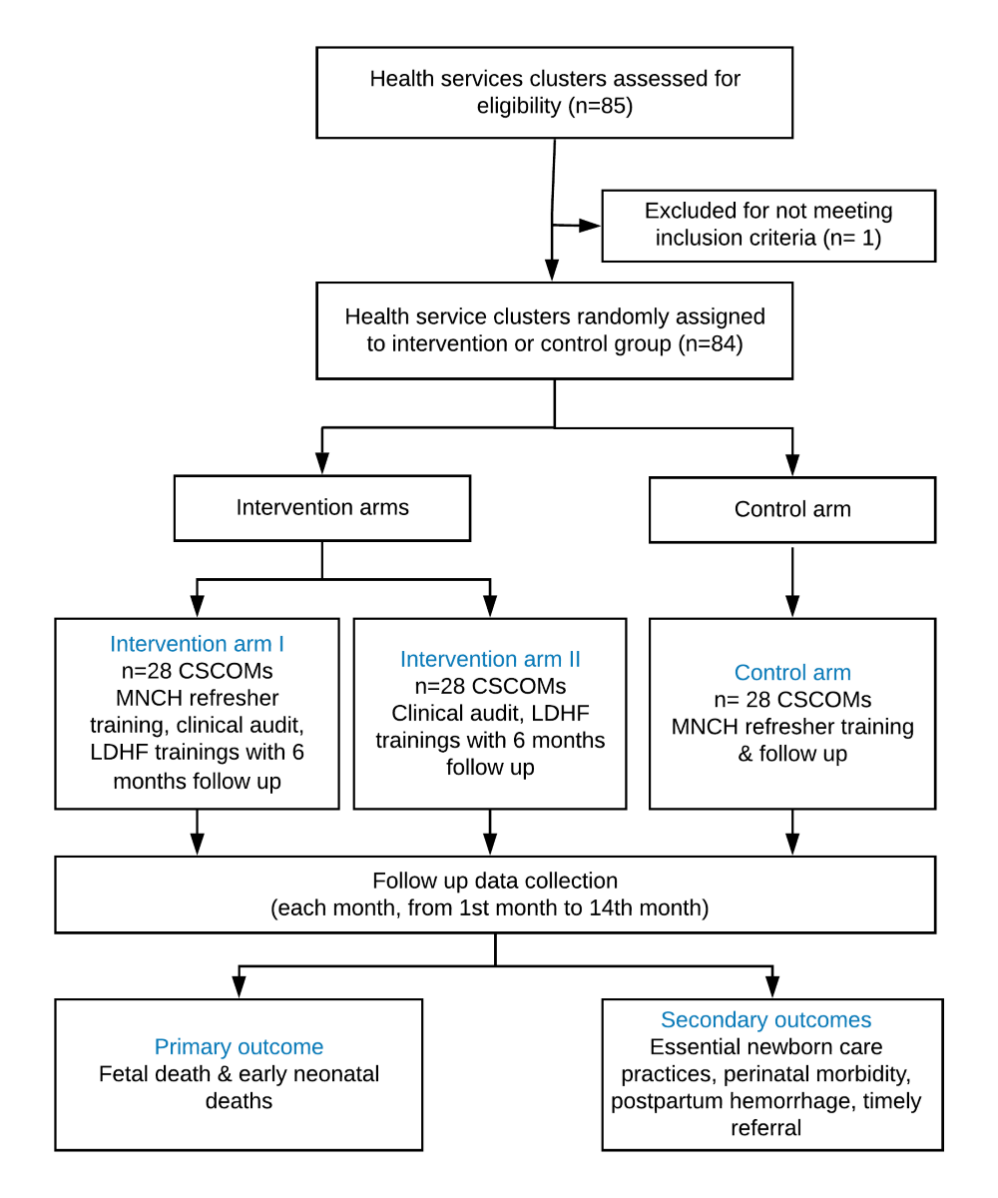
Flow chart of community health services clusters recruitment and follow-up. CSCOM: Centre de Santé Communautaire; LDHF: low-dose, high-frequency; MNCH: maternal neonatal and child health.

Health facilities were randomly allocated into one of the three study arms, resulting in 28 facilities per study arm. The randomization of clusters was performed by an independent epidemiologist not directly involved in the research project using a pseudorandom number sequence generated using Excel (Microsoft). Owing to the nature of the intervention, blinding of study participants and data collectors is not possible; however, outcome data will be collected by independent data collectors not involved in the intervention delivery. The study setting (Figure 1), recruitment strategies, random allocation sequence, intervention implementation, and data collection points (Figure 2) are summarized below.

### Trial Participants and Units of Randomization (Clusters)

The group of facility-based health workers and community health workers within the 4 health districts of the Koulikoro region within each facility (unity of randomization) are eligible to enter the trial. Eligible facilities are those that provide basic obstetric care and have at least one facility-based health care provider trained in AMTSL, ENC, and PNC. The eligibility assessment of facilities was made possible through the data collected during the baseline facility assessment conducted in December 2016.

Facilities within the 4 health districts of the Koulikoro region that did not provide basic obstetric care or those who did not have any health workers who received training in maternal and neonatal health care providers in AMTSL, ENC, and PNC have been excluded. Rural maternities were not eligible, as they are not included in the national audit policy.

### Intervention Strategy

The intervention uses a problem-based learning approach that uses the information emerging from the implementation of maternal and newborn audits to improve health worker skills training. This training is delivered by a study-trained facilitator, employed by Mali’s Ministry of Public Health and Hygiene, that attends and provides feedback during the audit meeting, followed by an immediate (next day) one-day in-service case-based focused training [8,13,14]. Specifically, each training cycle starts with the implementation of a maternal and neonatal morbidity and mortality audit, during which the trainer identifies gaps in knowledge and skills. This is followed by on-site LDHF hands-on training of maternal and neonatal health care providers on the skills and competency gaps highlighted by the audits. The training sessions also include direct observation of care, supervision, and use of simulations. In addition, community health workers are also trained when issues identified during the audit are related to their practice, such as in the identification of danger signs in the community or timely referral to the facility.

**Figure 3:**
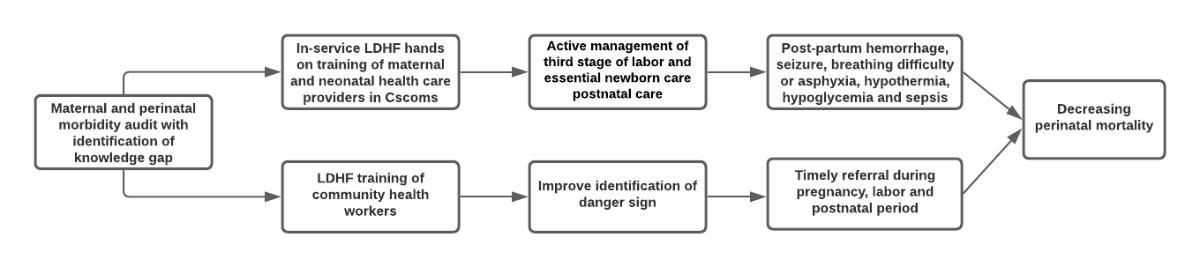
The intervention model. LDHF: low-dose, high-frequency.

We hypothesize that the provision of on-site case-based LDHF hands-on training of maternal and neonatal health care providers, focused on gaps identified through clinical audits, will improve quality of care, thus decreasing adverse events during labor and the immediate postpartum and perinatal period in facility births, in comparison to traditional off-site generic training. The training of community health workers will improve community management via identification of danger signs and timely referral during pregnancy, labor, and the postnatal period (Figure 3). Ultimately, the aim of the intervention is to address key barriers to improving maternal and perinatal health by focusing on increasing the capacity of maternal and neonatal health care providers along with improving the recognition of danger signs and referral during the natal and postnatal period by community health workers.

### Study Arms

#### Overview

This study is a three-arm cluster randomized controlled trial including one control and two intervention arms with a gradual increase in the intervention components.

- MNCH refresher training and follow-up: the control arm will consist of standard MNCH refresher training delivered in the classroom using multi-method classroom training delivered as part of the larger Canadian Red Cross and Malian Red Cross project. For these training sessions, two maternal and neonatal health care providers from each facility randomized to the control study arm received 10-day classroom training on AMTSL, ENC, PNC, and integrated community case management of childhood illnesses.
- Audit and feedback with LDHF hands-on training and 6 months follow-up: the first intervention arm will include 28 facilities in which mortality and morbidity audits will be implemented by the study team, followed by one day of case-based focused LDHF hands-on training biweekly for 6 months.
- MNCH refresher training followed by audit and feedback combined with LDHF hands-on training and 6-month follow-up: the second intervention arm will include 28 facilities that will benefit from MNCH refresher training, in addition to mortality and morbidity audits implemented by the study team, followed by one day of case-based focused LDHF hands-on training biweekly for 6 months.

#### Consent to Participate

Written consent will be obtained from mothers or fathers of newborns at the follow-up household visit and for verbal autopsy. If the mother is not able to give consent, her husband or other adult family member will be requested to give consent to participate in the study. If the respondent cannot read, the consent statement will be read to them, and a thumb impression by the respondent in place of a signature will signify consent. The data collector will explain the risks and benefits to potential enrollees before conducting the verbal autopsy and administering the household questionnaire in the intervention and control clusters. Participants will be informed that they have the right to withdraw from the study at any time and that there are no penalties from doing this, and this will not in any way affect their ability to receive any health care services.

#### Ethical Approval and Protocol Registration

Ethical approval was obtained from the Sickkids Research Ethics Committee (Research Ethics Board number 1000060635) and from the University of Bamako’s Faculty of Medicine Ethics Committee. The project was registered at ClinicalTrials.gov (NCT03656237).

### Process of the Intervention Implementation

#### Audit and Feedback

An audit committee comprising a health facility manager, basic obstetric and neonatal care providers, and district or regional health department representatives will be formed in each facility, following the guidelines developed by Mali’s Ministry of Public Health and Hygiene. The health facility representative designated by the audit committee will be responsible for audit support, including providing medical records and supporting documentation to the audit committee, assisting in the development of the process, procedural walkthroughs, and responding to various inquiries to assist the committee with the development and documentation of the audits.

#### Low-Dose, High-frequency Training

Mali’s Ministry of Public Health and Hygiene’s 10 senior physicians, with gynecological or obstetrical background, will be trained on the implementation of the intervention and will support implementation of the audits, facilitate the process and discussions, and during the audit meeting, identify key knowledge gap areas for the subsequent on-site case-based focused LDHF hands-on training, mentoring, and supervision of MNCH care providers. In this regard, the intervention trainers will stay at the health facility for one consecutive day to implement case-based focused LDHF hands-on training to the facility staff involved in basic obstetric and neonatal care and to community health workers linked to each facility when appropriate, as described above. The training modules will be based on recommendations from the clinical audit and focus on strengthening the core competencies identified by the audit committee as the cause of death or complications in the patient. The trainers will also observe health care providers during service delivery and postnatal care and will use direct observation skills checklist to record identified gaps in clinical skills and knowledge and will assist the health care provider in implementing national service delivery standards and guidelines. The audits will be implemented following the Ministry of Public Health and Hygiene guidelines and will serve as a platform for the identification of cases for case-based, focused LDHF hands-on training. Data from the partographs and facility birth registers will be used to identify the case to be audited. Once identified, audit cases will be anonymized and summarized by the health facility representative. The case summaries will be discussed in detail, identifying causes of death or near miss, perinatal or maternal complications, and assessing the quality of care provided. The findings will be evaluated against existing service delivery standards and protocols. In addition, external factors that adversely affect patient outcomes, such as delayed recognition of danger signs during pregnancy or labor, delayed referrals, and delayed recognition of danger signs during the perinatal period, will be identified during the audit for community health worker training at the corresponding facility. Audit information is the basis for identifying knowledge or case management gaps among health care providers, knowledge-to-action gaps, and training requirements. The summary of each audited case will have a structured section in which the trainer will document action points for training and managerial support, and these will also be recorded in the minutes of the audit committee meeting.

### Research Questions

This cluster randomized trial aims to assess whether LDHF hands-on training, informed by maternal and neonatal mortality audits in routine clinical settings, reduces perinatal mortality. Secondary objectives include estimating the effectiveness of the intervention in improving maternal and neonatal care practices among health workers and the comparative cost-effectiveness of the case-based focused LDHF hands-on training, compared with traditional training.

### Primary Outcome

The perinatal mortality rate per 1000 live births occurring in each facility and the corresponding catchment area for the births that had taken place in the facility will be used to assess the effectiveness of the intervention. It is defined as the sum of *fetal deaths* (stillbirth), defined as the death of a fetus weighing 500 g or more, or of 22 weeks of gestation or more, occurring in health facilities, and *early neonatal death*, namely, the death of a live born within the first 7 days of life occurring in the facility or in the community. As health facility studies are not an appropriate source of data for calculating perinatal mortality incidence [24] unless all babies are born and stayed in a health facility for 7 days, the data on perinatal mortality will be extracted from both facility-level data and household survey questionnaires.

### Secondary Outcomes

The secondary outcomes are as follows:

1. Proportion of newborns stimulated or with airways cleared using a suction bulb, catheter or a bag and mask to resuscitate per 1000 facility live births.
2. Proportion of children breastfed within an hour of birth per 1000 facility live births.
3. Proportion of newborns that were given breast milk and no other food or drink for the first 7 days of life, per 1000 facility live births.
4. Proportion of newborns dried and wrapped immediately after birth, per 1000 facility live births.
5. Proportion of newborns immediately placed on the chest of the mother—skin to skin after birth per 1000 facility live births.
6. Proportion of newborns who were not bathed within the first 24 hours of life, per 1000 facility live births.
7. Proportion of all newborns who received all four elements of ENC: immediate and thorough drying, immediate skin-to-skin contact, delayed cord clamping, and initiation of breastfeeding in the first hour.
8. Proportion of neonatal hypothermia, per 1000 facility live births.
9. Proportion of newborns referred from CSCOM to a higher-level facility because of obstructed labor, low birth weight, hypothermia, or severe infection.
10. Proportion of newborn mothers who experienced postpartum hemorrhage in the facilities.
11. Proportion of deliveries referred to the higher-level facility from a primary facility because of postpartum hemorrhage, per 1000 deliveries.

### Sample Size

A sample size of 600 births per cluster was calculated to have 80% power to detect a reduction in the perinatal mortality rate from 30% to 27% live births, which corresponds to a 10% reduction in the perinatal mortality rate between intervention and control arms (α=5%, intracluster correlation of 0.003, and coefficient of variation of cluster size 0.9 and a 0.96 correlation of baseline measures with outcome). The number of clusters available per arm was 28. The expected average number of deliveries per month in each cluster was approximately 38, resulting in 16 months of follow-up. Two additional months of data collection are planned to account for losses and refusals and allow for adjustments in the analysis, resulting in a final average cluster size of approximately 675 births over 18 months.

### Data Collection Methods

#### Health Facility Data

All the facilities in the intervention and control clusters will be visited once a month to retrospectively and systematically collect detailed data from partographs and registries for all facility births and any newborn that was not delivered in the facility but was referred to the facility for PNC within the first 7 days following birth.

#### Household-Level Interviews

The data collectors will also conduct a follow-up home visit (during the monthly health facility data collection visit, described above) to all women who recently gave birth in facilities since their last visit to administer a short questionnaire after at least 7 days postpartum to capture any perinatal death, complications, or adverse events, the status of the newborn during the perinatal period, and the mother’s satisfaction with the health care during the facility delivery.

#### Verbal Autopsy in the Household

In the case of perinatal death in the household recorded during their monthly visits, the data collector will pay a visit after the 40-day bereavement period to conduct a verbal autopsy. Two local independent physicians will review each completed verbal autopsy and assign the cause of death using International Classification of Diseases 10th Revision. If the two physicians do not assign the same diagnosis, a third physician (adjudicator) will review the two causes of death and assign a final cause of death.

#### Process Data From Audits and Training

The intervention trainers will collect data on the implementation of audits and training, the training modules provided, and the data on the health providers’ skills, using a structured checklist to examine providers’ skills related to AMTSL, ENC, and PNC. To ensure the quality of the data and training, supervisors will work full-time to monitor the data collection and training process and to check the reliability and accuracy of data compared with facility registries. Given the length of the project and action of multiple nongovernmental organizations in the study area, we will prospectively collect data on the concurrent implementation of MNCH interventions and health care worker training in the study facilities that may affect the study outcomes.

All data will be collected on tablets using ODK Collect and then uploaded to the server in real time. We will use a monitoring tool to oversee, in real time, the process of intervention implementation for evaluation. The study monitoring data included the number of health providers in the health facility trained, the number of community health workers trained, and the number of on-site case-based focused LDHF sessions conducted.

### Data Management and Analysis

#### Blinding

Owing to the nature of the intervention, facility staff cannot be blinded to the study allocation. Independent data collectors not involved in the intervention delivery will collect data to minimize bias in data collection. The primary outcome data analysis will be blinded to the intervention status of the facilities.

#### Data Safety Monitoring

To assess the progress and help ensure the validity and credibility of the trial results, a data and safety monitoring board will be convened with 3 individuals, respectively an epidemiologist from the Hospital for Sick Children, an obstetrician gynecologist, and a pediatrician from the University of Science and Technology of Bamako. Interim analyses will be conducted during the course of the trial, based on accumulating data on efficacy, to help the data and safety monitoring board determine whether there is sufficient evidence to recommend whether to continue, emend, or terminate the trial or if there is already clear evidence that (1) the intervention is beneficial, (2) the intervention is either harmful or has little or no effect, or (3) the trial will likely be unable to detect the effect of the intervention, for example, because the incidence of perinatal mortality and morbidity is too low or because of the low follow-up rate of the mothers during the perinatal period.

#### Process Evaluation

As this study is a complex intervention delivered in the context of an operational research, we will undertake a mixed methods process analysis to provide policy makers and practitioners information about how and why the intervention was effective and how it might be replicated [25,26]. First, the process evaluation will explore whether the intervention is delivered as intended [27], both at the facility level (training) and the individual level (provider delivery). Second, process evaluation will allow exploring the mechanisms through which interventions affect perinatal mortality and morbidity. As the intervention may have different effects in different facilities even if implementation was identical [28], we will assess the variability in the implementation to provide an understanding of how contextual and factors related to health facility features could act synergistically to influence the intervention implementation and perinatal mortality and to identify the contextual factors most likely to lead to successful implementation in other settings.

The process evaluation will be conducted in both intervention arms using the study monitoring data, semistructured interviews with stakeholders (n=12) and focus groups with facility providers and community health workers. Health care providers will be asked about their experiences and satisfaction with the intervention and suggestions to improve care for the AMTSL, ENC, and PNC. Health care providers will be asked about their perception of how to strengthen and sustain this intervention and other efforts to improve care for the AMTSL, ENC, and PNC in Mali. These steps will also help identify barriers to and facilitators of intervention implementation. Details on the protocol of our mixed methods process evaluation will be published in a separate paper.

#### Economic Evaluation

We will undertake an economic evaluation to determine the incremental cost-effectiveness of each of the case-based focused LDHF hands-on training strategies in comparison to MNCH refresher training in a classroom setting. This assessment will be conducted from the perspective of the health system. The resources used to deliver the intervention, including staff, training, implementation, and monitoring costs, will be used to derive the costs. The efficacy measurement will be based on the primary outcome variable of this study, namely perinatal mortality with a one-year time horizon and expressed in terms of the number of healthy years of life lost because of premature mortality [29]. The cost-effectiveness of the intervention will be measured in dollars per disability-adjusted life-year (DALY) saved, and we will estimate the incremental cost-effectiveness ratios of each training strategy compared with traditional training methodology by dividing the difference in mean costs between groups by the difference in mean effects between groups measured by DALYs. We will compare the LDHF hands-on training group with the traditional training in terms of (1) costs incurred over the 12-month period and (2) DALYs. All costs will be adjusted for inflation using the Malian Consumer Price Index and presented in international dollars.

#### Interim Analyses and Stopping Rules

No interim analysis was performed. However, in the event of disruptive events such as conflict, natural disaster, implementation of similar interventions in the study area, or the deteriorating health and humanitarian situation, we will assess the implications of the study design, stop the study if necessary, and assess the implications on the feasibility of the design to produce evidence on the implementation and effectiveness of the intervention.

## Results

The trial is completed. The recruitment began on July 15, 2019, and data collection began on July 23, 2019, and was completed in November 2020. Data cleaning or analyses began at the time of submission of the protocol. As the randomization is performed at the health facility level, we will undertake aggregate cluster-level analysis comparing the proportion between treatment arms [30]. We will compare differences in the proportion of perinatal mortality between intervention and control groups using a chi-square statistic adjusted for the effect of clustering within health facilities [31] using a formula that incorporates the intraclass correlation coefficient as a measure of the extent of clustering [32].

The analysis and report of the intervention effect will be carried out in accordance with CONSORT (Consolidated Standards of Reporting Trials) principles and its extension for pragmatic trials [33].

We will perform all analyses using Stata version 16.1 software (Stata Corp).

## Discussion

### Study Dissemination Strategy

This operational research study aims to improve the practical knowledge of health workers to improve the quality of management of childbirth and PNC to reduce perinatal mortality. It is part of Mali’s ongoing efforts to achieve target 3 of the Sustainable Development Goals and whose results would be useful for potential replication or scale-up. We will valorize the findings by adopting strategies for dissemination and knowledge transfer. For this purpose, the results will be disseminated to the Ministry of Health and Health Care providers. We will also hold a deliberative workshop with project users and stakeholders to discuss the results and lessons learned from the implementation and effects of the intervention to provide guidance from the perspective of replication or scaling-up. In addition, we will disseminate the results among the scientific and political communities through publications in scientific journals and presentations at international conferences.

### Strengths of the Study

The distinctive feature of this intervention compared with the other LDHF interventions is that it adopts problem-based learning, including supervision and audits with feedback [14]. Thus, the audit component will identify knowledge gaps related to the maternal and neonatal care provided in the facilities and then identify the appropriate training modalities to correct deficiencies in the management of maternal and neonatal care around the delivery and in the postpartum period.

The analytical approaches will provide evidence about the benefits and costs of this pragmatic intervention to improve the quality of maternal and newborn care in Mali with the aim of reducing perinatal mortality. The results will provide policy makers and practitioners with crucial information to facilitate the translation of best practices in capacity strengthening for health care providers in the routine practice of maternal and child care services. Indeed, although LDHF hands-on training has been implemented and evaluated in some resource-limited settings with the aim of reducing maternal and perinatal mortality, previous studies have analyzed only a few outcomes [17,21].

Another distinctive feature of this trial is that it adopts a pragmatic and holistic approach, and the analysis will contain both qualitative and quantitative data collected over a period of 14 months. The trial will provide a better understanding of how LDHF hands-on training of health care providers and community health workers could complement routine care to improve maternal and child health outcomes in resource-limited settings. In addition, this trial occurs during routine health service delivery and will demonstrate the potential of routinely collecting health management information system data to analyze the effectiveness of a major public health intervention.

### Limitations of the Study

Despite these strengths, some limitations of this study need to be considered. The facility-level data collection instrument was developed based on existing consultation and delivery registries, as well as partograph forms. As data are not being collected by direct observation of health care providers but rather from the partographs and registries they fill out, we are also concerned with the completeness, reliability, and accuracy of data to be collected. To overcome this important limitation, refresher training on the use of partographs will be held in all health facilities that are part of the trial. This will strengthen the health care providers’ capacity to use the partograph and aim to improve data completeness and quality for this study.

Some data will be lost to follow-up if the newborn’s mothers are referred to higher-level health facilities. However, we estimate that this will be a minimal disruption to the trial, and we will report the number of these cases in our analyses. In addition, underreporting of infant deaths is usually greater for deaths that occur very early in infancy in low-income countries [24]. As the Ministry of Health is a stakeholder in this project, a strategy will be developed in cooperation with the study team to motivate health workers to improve data completeness, reliability, and accuracy. We will also triangulate mortality data collected in the health facility with the mortality data collected in the household surveys to assess the level of completeness and accuracy and to reduce the likelihood of underreporting of perinatal and maternal deaths in the facility. The data collector will also conduct a follow-up home visit, aided by community relays and volunteers, for all facility births to minimize losses to follow-up.

Finally, completeness and accuracy of recall, including age at death, may deteriorate with time and is also related to the skill and cultural sensitivity of the person carrying out the interview [24]. However, as the recall period was relatively short in this trial (<21 days), the risk of recall bias will be reduced.

## Appendix

Multimedia Appendix 1 Peer-review report by SickKids - The Hospital for Sick Children (Toronto, Canada).

## Abbreviations

AMTSL: active management of the third stage of labor
CONSORT: Consolidated Standards of Reporting Trials
CSCOM: Centre de Santé Communautaire
CSREF: Centre de Santé de Référence
DALY: disability-adjusted life-year
ENC: essential newborn care
LDHF: low-dose, high-frequency
MNCH: maternal neonatal and child health
PNC: perinatal care
