# Supplementary material for: Combined Clinical Audits and Low-Dose, High-frequency, In-service Training of Health Care Providers and Community Health Workers to Improve Maternal and Newborn Health in Mali: Protocol for a Pragmatic Cluster Randomized Trial
Source: JMIR Res Protoc. 2021 Dec 10;10(12):e28644. doi: 10.2196/28644 (PMC8709918; doi:10.2196/28644)
Supplement: Multimedia Appendix 1 [file resprot_v10i12e28644_app1.pdf]

SickKids®
THE HOSPITAL FOR
SICK CHILDREN

Research Ethics Board

Report on Scientific Peer Review

PLEASE NOTE – Reviewers must include all substantive issues and/or recommendations on this form, whether or not they have been provided verbally to the investigator.

PART A: GENERAL

Primary Investigator: Dr. Diego Bassani Co-investigator: Dr. Zulfiqar A. Bhutta

Co-investigator: ______ Co-investigator: ______

Co-investigator: ______ Co-investigator: ______

Project Title The impact of case-based low-dose high-frequency hands on training of facility health care providers and community health workers on maternal and newborn health in Mali, a cluster randomized trial.

Funding Agency Global Affairs Canada (Funds already available) Deadline Funding received March 2016 (must be utilized by March 2020)

Brief Description of Project (to be completed by reviewer)

The study is an RCT which aims to understand the impact of case-based low-dose high-frequency training of health care providers and community health workers on perinatal mortality and morbidity. To achieve this aim it has three arms (2 intervention, and 1 control), arm 1 includes clinical audits, case-based low-dose high-frequency trainings, and traditional training, arm 2 includes clinical audits and case-based low-dose high-frequency trainings, and the control group will receive only traditional multi-method class room training.

PART B: BUDGET:

A) Approximate Budget - Year 1 351 560 B) Is it justified in the application? Yes

C) Are the sums requested adequate? Yes D) Do the items reflect the actual costs of the research interventions, excluding interventions that are part of routine clinical practice? Yes

E) Is there a project contract or agreement (notice of award)? Yes

PART C: REVIEW

Is the hypothesis reasonable? Yes

Is the literature review appropriate? Yes

Is the research protocol clearly described? Yes Is the stated significance of the study plausible? Yes

Are the summary pages well prepared? Yes

Are the research methods likely to deliver results to the stated objectives? Yes

Is this study feasible? Yes If not, why? ______

Is the study likely to yield publishable results? Yes

What is your overall assessment of the application?

The application is well prepared and grounded in the literature. It is a scienitifcally sound hypothesis and the results from this study could provide valuable information that may have important implications for the training of health providers and community health workers in Mali and possible effects on perinatal mortality.

Please list any specific recommendations (attach an additional page if necessary).

1. Asked to highlight how far apart CHW catchment areas are from one another and possibility of diffusion of ideas/best practices between CHWs
2. How will it be ensured that MRC volunteers/data collectors are visiting households/completing forms
3. Previous studies that show the rate at which MRC volunteers capture births and deaths
4. Wanted more information about the fingerprinting for consent in Mali, if there were issues of mistrust of high-level institutions tthat would cause individuals to hesitate to give their fingerprints or if this was a common accepted consent procedure in Mali
5. Wanted more text in the ethics section about individuals being able to stop verbal autopsy or interview, and being able to skip troubling questions

Please see appended sheet for additional comments from Reviewer 2

PART D: FOR HUMAN RESEARCH ONLY

Which of the following prior studies have been published?

Relevant animal studies No

Studies of animals at a stage of development analogous to the subjects of the proposed study No

Relevant adult human studies Yes

If the answer to one or more of the above questions is negative, please comment on the feasibility and desirability of undertaking prior studies in animals and/or adult humans before proceeding to a pediatric study.

Animal studies are not relevant. This is a study focused on improving quality of care during childbirth with no interventions in the pediatric age group.

Are patient eligibility and exclusion criteria clearly delineated? Yes

Are the following methods appropriate? Yes

Ascertainment of potential subjects Yes

Making contact with potential subjects Yes

Obtaining consent (if needed) Yes

Is the study comparative? Yes

Are the study numbers discussed and justified? Yes If yes, are the study numbers sufficient to provide likelihood of an interpretable result? Yes

Are the subjects likely to be enrolled in other studies? No

Is the study descriptive? No If yes, is the information to be derived likely to be unique?______

Does the study involve disruption of schedules (including school) for subjects/parents? No

If yes, is the disruption justified? NA

Are the potential harms vs. potential benefits appropriate? (For research in children, potential harms must be estimated to be more than balanced by potential benefits; both are quantified in terms of the expected frequency of the harm or benefit, and the magnitude of the harm or benefit) Yes

If this is a clinical trial comparing two or more treatment regimens, are the risk - benefit ratios of each regimen well balanced so that the average expert would not favour one regimen over the other (ie., equipoise exists)?

Yes ______ No ______ Don't Know ______

Is statistical analysis required? Yes If yes, is there a discussion of statistical methods and are they appropriate? Yes

Is the plan for monitoring safety and efficacy (in the case of diagnostic or therapeutic trials) of the human subjects appropriate? NA

For clinical trials (diagnostic as well as therapeutic), is the plan for monitoring safety and if relevant, efficacy, appropriate? (see attachment for categories of research and the monitoring matrix) NA

Should extra-mural scientific peer review be obtained (e.g., for conflicts of interest of the researchers, the institution, and or the internal peer reviewers; for questionable risk - benefit ratio; for serious threats to the privacy of human subjects)? No

Are there any major changes that need to be made before this proposal should be submitted for ethical review?

No major changes need to be made to the proposal before it is submitted for ethical review.

Assuming that this committee will accept any changes made to the protocol, is scientific merit including significance of the study adequate to justify its ethical consideration? Yes

PART E: RANKING

Please rank the proposal as is, and the proposal if the proposed revisions are made.
Please use the two digit CIHR rating system: 4.5 - 4.9 outstanding, 4.0 - 4.4 excellent, 3.5 - 3.9 very good, 3.0 - 3.4 acceptable, but low priority, 2.5 - 2.9 needs revision, 2.0 - 2.4 needs major revision, 1.0 - 1.9 seriously flawed, 0 not acceptable.

Reviewer	Signature	Scientific Discipline	Proposal As Is	Proposal after Revisions Made
Anushka Ataullahjan		Public Health	4.3	4.4
Lisa Pell		Global Health	4.3	4.4

Date of Review: January 3, 2018

disciplines involved in research	disciplines of investigators	disciplines of scientific reviewers
public health	public health	public health
	epidemiology	global health
	pediatrics	

PART F: ITEMIZED RESPONSE

An itemized written response to all the issues raised by the reviewers noting where revisions were made in the revised protocol must be provided to the Research Committee Review Chair/Grant Review Committee Chair for final approval & signoff prior to submission to the REB for ethical approval.

Final Approval of Research Director/Committee Chair: ______________
Date: Jan. 17, 2018

PART G: CONFLICT OF INTEREST DECLARATION (for reviewers):

Please confirm with your signature that all contracts and any conflicts of interest (actual, apparent, perceived, or potential)* relating to this project are disclosed to the Manager, Research Contracts for review.

Signature______________

* Conflicts of interest include but are not limited to the following situations:
Do you or any of the involved staff members or your/their dependents have,
(1) employment or consulting arrangements and/or a financial interest in the sponsor of the study, or with proposed subcontractors, vendors, or collaborators;
(2) a financial interest in the product/medical device that is the subject of the study?

IPR-Additional comments from Reviewer 2:

General Comments

- The study rationale is strong and supported by scientific evidence. Given the high rates of perinatal mortality in Mali, there is a great need for this impact-focused trial.
- Strong partnerships are in place with in-country organizations and the team at SickKids has an abundance of experience in conducting and evaluating clinical trials in LMICs. The strength of the team bolsters the feasibility of this study.
- Given the pragmatic trial design, pending results of the trial, there is a high likelihood that this work will scale and be self-sustaining.
- To improve the proposed protocol and study materials, I would suggest that you consider the following:

PROTOCOL

- Provide greater clarity on how the Mali audit system works. You repeatedly mention that it is mandatory to "notify maternal, perinatal, and neonatal deaths/adverse events within 48 hours and to convene an audit of the same within 15 days..." but you don't make it clear a) who is making the report, and b) to whom the notification is being sent. Also, what, if any, systems are in place to ensure that these processes are being followed?
- Will stillbirths that happen at home be captured? Please review your inclusion criteria listed in section 12 of protocol. The way it's currently written, it does not sound like stillbirths at home will be captured/included in the outcome. The criteria refers only to 'Live home births' and 'all CSCom deliveries" so what about non-live home births?
- Provide greater clarity on when/where consent will be collected. Will consent be collected at the facility for those women who deliver at a CSCom and at home at day 8 follow-up visit for those women who delivery at home? Will the same consent form be used for everyone? Or will there be a difference consent form based on trial arm assignment? Who on the study team will be responsible for collecting consent?
- Provide greater clarity around the study's analytical plan. Will you adjust for clustering or analyze at the level of the cluster? If adjustments will be made, how will you do it? Do you plan on performing any per protocol analyses? If so, you should state these a priori (to avoid issues at time of publication) and clearly define what will be considered 'per protocol'. How will you deal with missing data? How will verbal autopsies be analyzed? Who will review them? What algorithms will be used to assign cause of death?
- Provide more information on the role of the DSMB. How many times will they be provided data? Will the results be blinded (no arm info. provided)? What are the stopping rules? Will the DSMB meet virtually? If so, how many times? The protocol states that Dan will be the SickKids representative on the DSMB. Do you still need me to participate?
- How will one data collector move between 14 facilities? What's the proposed mode of transport? Will you provide reimbursement for these transportation expenses? I don't see this in your budget and this concerns me about the feasibility of data collection.
- Review the protocol for minor grammatical errors including the misplacement of commas, semi-colons, colons etc.

QUESTIONNAIRE

- A verbal autopsy tool was not provided for review. This will need to be prepared and submitted to the REB.

- Will you have a separate consent form that is not embedded into a questionnaire? Will there be a separate consent form for each arm of the trial? I believe that a separate consent form should be uploaded to the REB.
- Questionnaires collect information about participant name (newborn and mother). Unless you can justify this, please remove as the REB will not allow these data to be collected.
- Add a version date/number to each questionnaire - required by REB.
